# Breaking the Mould: A Theory Explaining How Young Adults Living with WS Learn Life Skills through Music

**DOI:** 10.3390/ijerph19010049

**Published:** 2021-12-21

**Authors:** Ewie Erasmus

**Affiliations:** Musical Arts in South Africa: Resources and Applications (MASARA), School of Music, North-West University, Potchefstroom 2520, South Africa; Ewie.Erasmus@nwu.ac.za

**Keywords:** Williams syndrome, life skills, inclusive music education, wellbeing, compassionate music education, adult education

## Abstract

This article presents a theory explaining how young adults living with Williams syndrome (WS) learn life skills through music. The article answers the question: What theory explains how young adults living with WS learn life skills through music? The theory presented in this article is informed by principles of care ethics and compassionate music education and theories of wellbeing and flourishing. The theory is further informed by empirical data, including data collected during semi-structured interviews, casual conversations, observations, field notes, and social media and blog posts collected at Berkshire Hills Music Academy, Massachusetts, USA, over six weeks. Thematic analysis was the data analysis strategy. The theoretical proposition represented by the findings is: (i) If young adults living with WS have the opportunity to learn through engagement in music activities within a safe environment in which they are engaged, supported, appreciated, motivated, feel that they belong and feel that they are competent, and (ii) if educators are willing to focus on the abilities of these young adults by putting their needs first, (iii) then young adults living with WS should be able to overcome various challenges and ultimately develop the life skills they need to live well.

## 1. Introduction

It is said “the deeper the mud the lotus grows in, the more beautiful its flower” [1], p. 32. Robin L. Brooks [1] uses the image of a lotus flower as a symbol of hope, joy, fulfilment, and light when writing about her journey towards self-love, finding her self-worth, and healing after childhood abuse. Similarly, Bruce Logan [2] explains how lotus flowers emerge as bright, pure, and beautiful flowers despite growing from muddy waters. He writes about how these flowers rise above adversity while being washed clean by the murky water they rise from. In Buddhist practice, the lotus flower symbolises enlightenment and represents the notion that humans need to overcome suffering and difficult times if they are to reach spiritual enlightenment [3]. Lotus flowers further represent love, compassion, and the ability to rise out of suffering [4].

This study was inspired by my personal experiences as a music educator in secondary and tertiary education, my passion for advocating for access to quality, meaningful music education, as well as previous experiences with individuals living with WS [5,6]. This article presents a theory explaining how young adults living with WS learn life skills through music. This theory could heighten awareness of the importance of following strengths-based, abilities-first approaches to music education which are informed by wellbeing theories [7,8], ethics of care [9], and compassionate education [10]. It is paramount that educators, therapists, and society at large care for the unique needs of all individuals if we are to accommodate not only their assumed needs but their expressed needs [9] within our communities and the education system [11]. Consequently, adult educators and curriculum developers need to work with young adults when planning learning programmes to, as mud and water provide nourishment for the lotus, design curricula and learning environments that enable these young adults to flourish. Education policies need to encourage “the creation of relationships of care and trust between teachers, teachers and students, students and students” [9], p. 16. It is further important to advocate for teaching strategies that prioritise attentiveness, responsiveness, and wellbeing needs [11,12].

Compassionate music education promotes a focus on individuality which supports an abilities-first, strengths-based approach to teaching and learning. Compassionate music education also acknowledges shared humanity and prioritises emotional virtues related to kindness, care, empathy, sympathy, and a response to the suffering of others [10,13]. Furthermore, compassionate education is related to an ethic of care which supports notions that educators should care for and about [9] the needs of young adults living with WS and that they must listen for and attend to their unique needs. Noddings [9], Hendricks [10], and Peterson [13] similarly speak about an awareness of others’ suffering, relationships of trust and providing support. Hendricks [10] warns that teachers often mistake pity for compassion, adding that it does not serve to meet educational needs if teachers place themselves in a superior position. When Hendricks [10] writes about compassionate music teaching, she proposes that teachers support and guide students through a shared enthusiasm for music and that teachers should support students’ desires and passions. Therefore, when we approach music education in a compassionate way, we should aim to be more focused on addressing our students’ unique, individual learning needs. Consequently, we need to shift our focus from teaching approaches merely seeking to improve skills or fix problems among our students. Instead, we have to connect with our students and build on their strengths, abilities, and interests through compassion and care. In this way, young adults, including young adults living with WS, can gain the life skills that they need to flourish within their communities.

WS is a neurodevelopmental genetic disorder that occurs when roughly 20 genes are deleted from chromosome 7 during conception and affects 1 in 10,000 people worldwide [14,15,16]. The syndrome is associated with distinctive physical features (including a wide mouth, small, often widely spaced teeth, elf-like ears). WS is also associated with a specific neuropsychological profile that influences the behaviour of those diagnosed and leads to specific strengths (language and music) and weaknesses (mathematics, visual-spatial skills), medical manifestations (including cardiovascular abnormalities, hypercalcemia, and developmental delays) and high sociability. [15,16,17,18,19,20,21,22]. Individuals living with WS are drawn to music [23,24] and have even been described as being “consumed by their affective reactions to music” [20], p. 3. Researchers have further confirmed that people diagnosed with WS experience extreme emotions when they engage with music [23]. In addition, these individuals often display musical abilities above what one would expect when considering their cognitive abilities [19,20,25,26,27]. The love for and attraction to music that these young adults display [20,23,24], along with my passion for advocating access to quality, meaningful music education for all, led me to explore the possible importance of music in these young adults’ learning. Although it is not true that all individuals diagnosed with WS are necessarily musically gifted, one could say with confidence that it is more likely that these individuals will engage in music activities than people without WS [19,20,24]. It also seems that individuals living with WS are very attentive to music [24] and that music helps these individuals cope with specific challenges while reducing their anxiety levels [6]. Further, music and the arts have been found to enhance the learning processes of individuals in the general community and specifically to meet the needs of those who have intellectual disabilities [25,26,27,28]. Studies by Reis et al. [29] and Biel [30] emphasise the importance of music in the learning process of individuals with WS, specifically attaining non-musical goals. A study by Ioannidi and Samara [31] revealed that when children living with WS have the opportunity to learn through music, they can grasp non-musical concepts more easily and consequently overcome certain cognitive and personal difficulties. Yet, the notion of learning though music is still uncharted territory where life skills education for young adults living with WS is concerned. Furthermore, these young adults do not have sufficient access to post-secondary institutions that can address their distinctive educational needs, especially when seeking to develop basic life skills through music as young adults.

**Figure 1:**
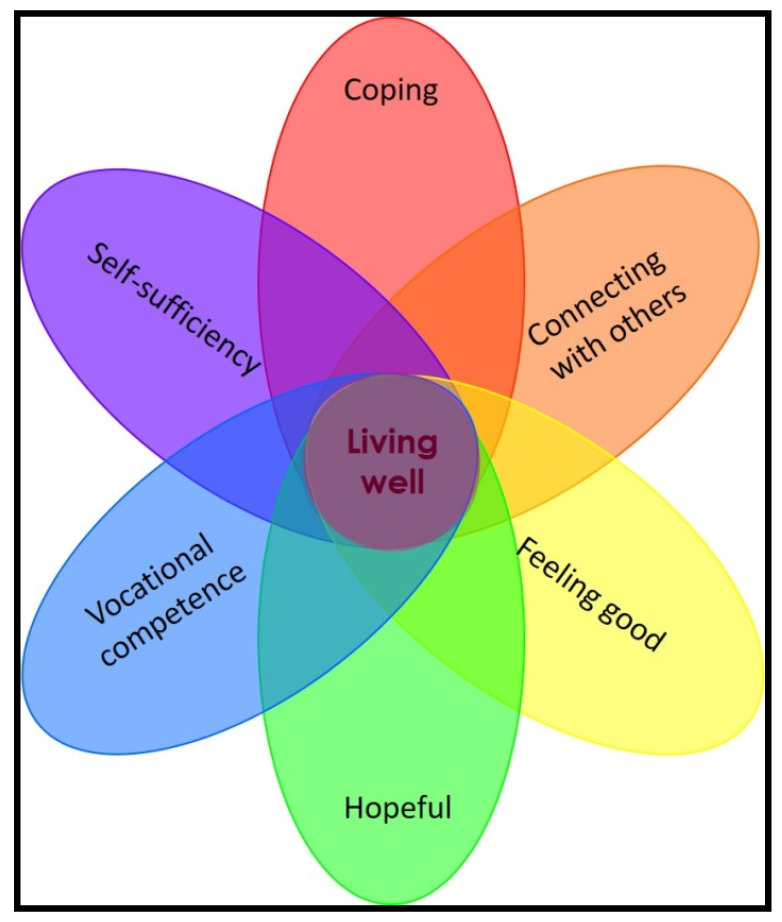
A conceptual framework, grounded in the scholarly literature, explaining what life skills young adults with disabilities need to acquire.

According to Hodge et al. [32], scholars have used terms such as “social-emotional learning, emotional intelligence, positive psychology, resilience, and character education” (p. 1127) as synonyms for life skills. Life skills generally include skills associated with cognitive abilities, personal and interpersonal skills [33,34], the ability to cope with anxiety, problem-solving skills, and the level of life satisfaction that an individual experiences [35]. Educational programmes focused on teaching life skills therefore aim to support individuals in the development and application of self-management and psychosocial skills with the aim of positive behavioural change [33]. Although life skills as a concept is often defined as the ability to cope with everyday challenges [35,36], identifying the specific skills needed to achieve this level of coping is contested. This problem is due to the notion that the nature of life skills as well as individuals’ ability to cope with various challenges are context-specific and consequently determined by various cultural and social factors [32,34,36]. It is, therefore, important to keep in mind that the specific life skills that a person requires might differ depending on the context in which they find themselves as well as the challenges that they have to overcome at any given time. For the purpose of this study, after an extensive review of the literature, seven life skills were identified. These life skills reflect the skills that young adults living with WS and other disabilities have to acquire to be able to flourish and cope with everyday challenges. The life skills are represented in a conceptual framework that the researcher developed as part of this study (Figure 1) and include:

1. Coping with various everyday challenges (including, among others, stress, change, managing emotions, setting and attaining goals, and time management) [34,35,37,38];
2. Connecting with others and fostering meaningful relationships [8];
3. Feeling good and managing emotions (also including the ability to experience life satisfaction and to be confident) [8,39];
4. Being hopeful [8];
5. Developing vocational competence [40,41];
6. Being self-sufficient [42], and;
7. Living well [8,37,41].

Music and the arts have been found to enhance the learning processes of individuals in the general community and, specifically, to meet the needs of those who are intellectually disabled [25,26,27,28]. Yet, the notion of learning through music is still uncharted territory with regard to life skills education for young adults living with Williams syndrome. Music seems to be one of the few ways in which these young adults can make sense of their educational experiences in order to acquire life skills [6,29,30]. A study conducted by Stickley et al. [43] also suggests the importance of arts-based instruction, as they found that intellectually disabled individuals experienced personal and social development through participation in arts activities. The study further revealed that, through the arts, these individuals were included in their communities by being able to overcome various social barriers that they had previously experienced. Music could be used not only as a means for acquiring the life skills needed to connect with others and cope with everyday life [44,45], but also provides an opportunity for young adults living with WS to learn skills that would ultimately permit them to become independent and live fulfilling lives [29,30]. A study by Erasmus and Van der Merwe [6] revealed that one of their study participants specifically mentioned that music helps him to learn more efficiently. Studies by Biel [30] and Reis et al. [29] revealed that music provides unique opportunities for children living with WS to learn non-musical skills. Consequently, for many young adults living with WS, music is one of the only ways in which they can acquire the life skills needed to become independent and cope. In their study, Reis et al. [29] found that young adults living with WS had positive learning experiences when the Music and Minds programme followed a talent development and abilities-first approach to address divergent learning needs through a focus on musical talent. Biel [30] also argued that music could be the key to successfully educating individuals diagnosed with Williams syndrome. This success can be ascribed to the fact that music provides a fun, manageable learning experience for most individuals who live with specific disabilities. This notion that music may well be the key to learning for individuals living with WS is central to my study, as I contend that educational programmes that focus on talent development and enrichment through music could allow young adults living with WS to learn optimally.

The affinity that young adults living with WS display toward music [15,16,19,20,21,24,46,47,48,49], as well as the notion that these individuals could stand to benefit greatly when learning life skills through music [5], strengthens the argument for the necessity of this study. According to Biel [30], individuals with WS expressed frustration with learning in general and said that they wanted to be normal. This notion of struggling to learn is in line with studies by Erasmus [5] and Erasmus and Van der Merwe [6] which also found that individuals diagnosed with WS longed to be normal, to be included into their communities and to feel accepted, and that music provided them with such an opportunity. When educating individuals with WS through music, one could provide them with an environment in which they experience less frustration while learning, while also experiencing acceptance from their peers and a sense of belonging [29].

Educational programmes focusing on talent development and enrichment through music could consequently allow young adults living with WS to learn optimally. Educational programmes structured around music, talent development, and principles associated with an abilities-first approach could enable these young adults to cope with challenges in everyday life, while also avoiding the paternalism and pressure that comes with an educational focus on deficits and the fixing of certain problem areas. Music could consequently be used not only as a means for acquiring the life skills needed to connect with others and to cope with everyday life, but also to provide young adults living with WS with a fun, manageable opportunity to learn skills that will ultimately permit them to become independent and live fulfilling lives [44,45]. For many young adults living with WS, music is one of the only ways to acquire the life skills needed to become independent and cope with everyday challenges. Therefore, the purpose of this study is to generate a theory that explains how young adults with WS learn life skills through music.

## 2. Method

This qualitative study follows an instrumental, theory-building case study approach and is informed by social constructivism [50]. The phenomenon of how young adults living with WS learn life skills through music was consequently studied in real-life contexts at BHMA and during community-based performances. During data collection and analysis, the author focused on processes, motives, causes of behaviour, and personal values [50,51]. This case study consequently studied a contemporary phenomenon in everyday contexts that are influenced and determined by context-specific conditions. The qualitative design of this study allowed the researcher to report on the participants’ voices by gaining access to their personal views [51,52].

According to Anfara and Mertz [53], a theory consists of concepts, constructs, and propositions, with the links between the concepts and constructs representing the theory. In this case the themes and categories emerging from empirical data analysis are the concepts, as well as the findings emerging from a review and thematic analysis of literature in the fields of music education, music therapy, community music, community music therapy, inclusive education, wellbeing, care ethics, compassionate music education and life skills education. The constructs are the five theoretical components [50,51,52] which guided the data analysis process and explain how young adults with WS learn life skills through music. These theoretical components, based on the findings emerging from this study, include: (1) Central phenomenon and context: Engaging in music activities facilitates learning; (2) Causal conditions: Optimal circumstances for learning life skills; (3) Strategies: How BHMA staff facilitate life skills; (4) Intervening conditions: Conditions inhibiting learning for young adults with WS at BHMA; and (5) Consequences: Life skills.

### 2.1. Research Approach for Collecting and Analysing Empirical Data: Instrumental Case Study

The empirical data for this study were collected by following an instrumental case study approach to explain the identified phenomenon within a real-life context and bounded system [50,52,54]. Consequently, the aim of this instrumental case study was to gain a deeper understanding of how young adults living with WS learn life skills through music (phenomenon) at BHMA (bounded system). Furthermore, an instrumental case study approach provided the opportunity to expand on existing theories and to generate a new theory on how these young adults learn life skills through music.

#### 2.1.1. Identifying the Case

BHMA offers a post-secondary transition programme through a Two-Year Certificate Program focused on teaching life skills, communication skills, vocational skills, and music to young adults who are intellectually divergent. The programme aims to prepare these young adults for independent and community living. BHMA also offers a programme where graduates of the Certificate Program can become LIVE members (Long-Term Independent Vocation Experience ensemble groups for students who choose music as their vocation after graduating from the two-year programme) while following programmes specifically tailored to their individual needs, or whereby they can join the Performance Troupe (an ensemble group for more advanced musicians who finished their two-year vocational programme). During my six-week visit to BHMA, I focused on young adults with WS who are part of the LIVE programme or the Troupe. BHMA was identified as the site for data collection as it is the only institution of its kind, as far as I am aware of, that focuses on meeting the needs of young adults living with WS through music. When the academy opened its doors in 2001, 85% of the students enrolled had WS. The idea of a music academy focusing on special needs was coined after several music camps were held by the Williams Syndrome Association over 10 years and the Music and Minds programme was hosted by the University of Connecticut in 1998–1999.

#### 2.1.2. Data Collection

Data were collected over six weeks at BHMA and include photos, videos, notes on participant observation, transcripts from in-depth, semi-structured interviews, and transcripts of informal conversations with BHMA staff and students attending BHMA. During the six weeks, the researcher observed between 12 and 15 classes per week. During observation, the researcher focused on the interaction between participants and participants and staff, relationships, processes during the learning process, the type of skills that are being facilitated, participant reactions, behaviour, and routines. These classes included ensemble practice, choir practice, dance, and individual music lessons. Weekly Variety Hour concerts were also observed on Friday afternoons. During the concerts, young adults attending BHMA had the opportunity to share their skills and talents with BHMA students and staff, family, and community members. I also accompanied various ensemble groups on community-based performances on Wednesdays. Interviews with music educators and music therapists at BHMA and with parents of young adults living with Williams syndrome lasted between 40 min to 1 h. Staff member Liz participated in two interviews, and we further had various conversations of which I took notes. My conversations with BHMA students Thorny Rose and Tori lasted roughly 30 min and Tori responded to questions via email at a later stage. Data further include posts from the BHMA public Facebook page and blog [51,54,55,56]. The participants for this study were purposefully selected and had to be willing and able to provide a unique, in-depth understanding [50,51,54] of how young adults living with WS learn life skills through music. The participants for this instrumental case study include 2 music educators and 2 music therapists at BHMA who work with WS students, 11 students living with WS enrolled at BHMA, and the parents of 3 of these students. The young adults living with WS who participated in this study include 4 males and 6 females between the ages of 24 and 35. All of the young adults completed secondary school and have Individualised Education Plans, ensuring that their unique needs are met.

#### 2.1.3. Data Analysis

Data were analysed through thematic analysis in ATLAS.ti 8. During data analysis, process coding was utilised to focus on routine, activity, actions, behaviour, time, and sequence [57,58,59]. Data analysis was an emergent process and was also initially informed by the conceptual framework for life skills (Figure 1) which provided some in-vivo codes guiding the initial analysis process. The data analysis strategy involved an exhaustive comparison between small units of text. Through thematic analysis, emergent categories, and themes, grounded in the data, the researcher was allowed to develop an interpretative understanding of how young adults living with WS learn life skills through music at BHMA. The emergent themes and categories were shared with another researcher to ensure transparency and credibility. Findings were also shared with the participants. Concept-mapping further offered a means to visually represent the data, which assisted in the conceptualisation of theory [50,51,52,53,57,60]. Ultimately, the six steps of data analysis defined by Clarke et al. [59] were followed: (1) familiarising myself with the data by reading and reviewing the data; (2) coding the data through process coding [57]; (3) searching for themes by identifying similarities and relationships between codes and categories and searching for meaning in the data; (4) reviewing the themes to ensure that the themes correlated with the quotes assigned and to the data; (5) describing each theme with a description of the boundaries of the theme and capturing the theme’s essence; and (6) writing up the findings through a process of critical reflection. After the final themes were identified, theory was conceptualized by drawing on Corbin and Strauss’s [61] axial coding paradigm. The process of theory development was consequently informed by five theoretical constructs [50,51,52] (see Section 2) and emphasising the relationship between each of the constructs. This relationship between the constructs ultimately informed the theoretical proposition presented in this article and informed the structure of the results section.

### 2.2. Ethical Considerations and Trustworthiness

The BHMA board of directors granted permission to collect data at BHMA before potential participants were contacted. All the participants completed informed consent forms and ethical clearance was obtained to conduct the study. The findings that emerged from this study were further shared with participants to ensure that the conclusions that were drawn accurately represent the participants and BHMA. All the names included in this article, apart from BHMA (with permission from stakeholders), are pseudonyms. The trustworthiness for this study was ensured through prolonged engagement, an intimate knowledge and understanding of the research topic and context, and multiple sources as evidence during data collection [50,51,52,62,63].

## 3. Results

**Figure 2:**
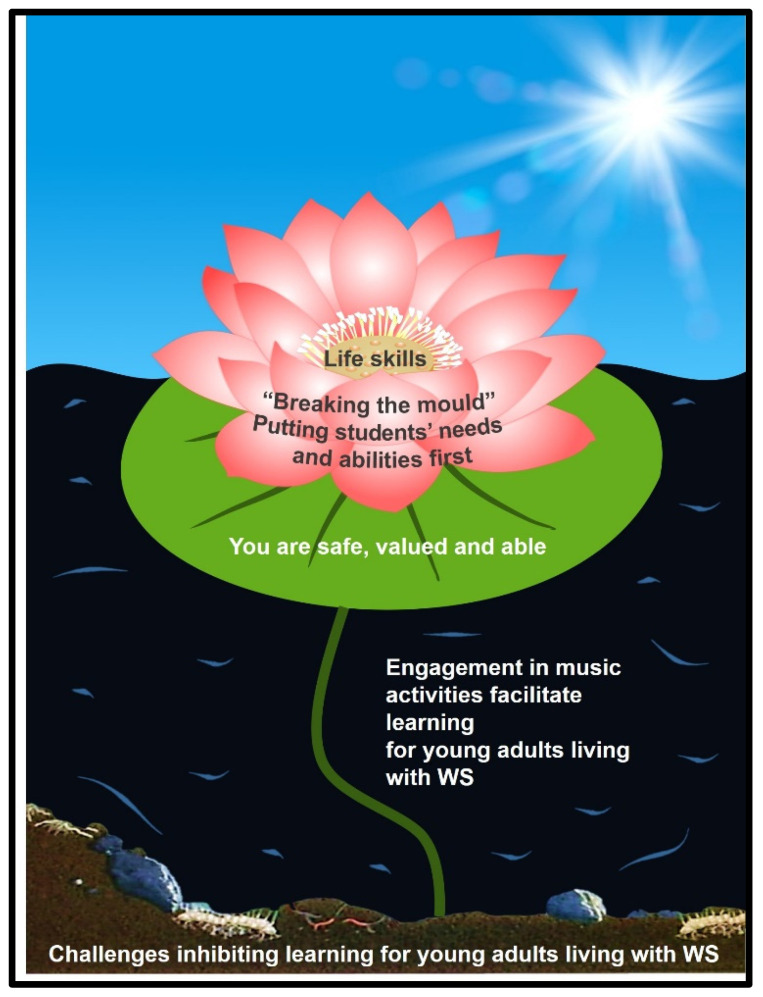
A theory explaining how young adults living with WS learn life skills through music.

The findings presented in this section are informed by five theoretical components: (1) central phenomenon and context (engaging in music activities facilitates learning); (2) causal conditions (optimal circumstances for learning life skills); (3) strategies (how BHMA staff facilitate life skills; (4) intervening conditions (conditions inhibiting learning for young adults with WS at BHMA); and (5) consequences (life skills) (Figure 2).

### 3.1. Central Phenomenon and Context: Engaging in Music Activities Facilitates Learning

At BHMA, young adults living with WS learn life skills through engaging in music activities which include (1) individual lessons, (2) participating in music making with others by moving with others and being part of ensembles, and (3) music performances. The “enduring connection” that these young adults have with music is at the core of the BHMA curriculum. For Horn Man’s mother, Mrs Ellers, the “sharing of music is important” and is an integral part of the success of BHMA. Liz and Johann, both BHMA staff members, emphasised that engagement in music activities is a key factor in the acquisition of life skills for young adults with WS at BHMA. Johann further stated that “music is truly the one factor that ties our sessions with WS students together” and that music “tremendously accelerates their learning process”. When speaking about the importance of music in his life, Allan said that “(m)usic is very important because it has a big impact in my life, and it helps me learn”. During my time at BHMA, and from observing and listening to the staff and the young adults with WS attending the Academy, it became clear that without engagement in music activities, these young adults might not acquire life skills as efficiently and successfully. For the young adults with WS at BHMA, engaging in music activities at BHMA and in the local community not only acts as a motivator to learn and step out of their comfort zones, but also facilitates their learning.

During the six weeks that I spent at BHMA, I witnessed how Tammy’s confidence and sense of self improved as a result of the support and encouragement that she received during her individual lessons. For Angela and Thorny Rose, individual lessons provide opportunities to work on vocational skills and to learn repertoire that enables them to express themselves and share their stories through music. Most of the ensemble sessions at BHMA are rehearsal opportunities, apart from the times when these young adults rehearse for their individual lessons. Rehearsal is consequently an integral part of the BHMA curriculum, as it encourages students to be “engaged in the music activity and to be present in practising and rehearsing” while working towards “life skills and social skill development”.

### 3.2. Causal Conditions: Optimal Circumstances for Learning Life Skills

I believe that the learning environment that the founders envisioned and that BHMA staff have created over the years plays a vital role in the way that young adults living with WS enrolled at the Academy acquire life skills. During data analysis, seven causal conditions emerged as being integral to how the BHMA programme is structured. The success of the BHMA programme is, therefore, partially determined by the fact that young adults with Williams syndrome at BHMA (1) have the opportunity to learn within a safe environment, (2) are supported, (3) feel appreciated, (4) experience a sense of belonging, (5) feel motivated to learn, (6) are engaged in the learning process, and also by how (7) BHMA staff have managed to create a space in which young adults living with Williams syndrome can feel competent.

When I spoke with Liz, a staff member at BHMA, about their programme and their vision, she said that “helping the students have a safe place is the first piece that we try to create, and I think we were successful in creating (this) years ago”. She also stated that “if the environment in which they are learning and living is safe, then their resistance or their fight-or-flight response isn’t engaged” and, therefore, their “ability to absorb that incoming information is that much more present”. It thus became clear that it is one of the top priorities of the BHMA staff to create an environment in which young adults with Williams syndrome can “feel comfortable” and safe while they learn, and that creating this type of environment ultimately promotes optimal learning for these young adults. Just as Liz spoke about young adults with WS needing to feel supported and safe, she also said that “the second piece was respect, and creating an atmosphere here where the students feel respected and that they’re valued”. Liz specifically mentioned, with reference to young adults with WS, that “the connection to music really helps initiate (their learning)” and that an environment where they feel appreciated “is really where the learning and the growth and the blooming happens”. She supports this by stating that “you can get very clinical about it or very not clinical about it, but I think the core of it all, and from a psychology perspective, the core of the human nature is wanting to feel valued”. Data analysis confirmed that BHMA staff were successful in creating spaces where young adults with WS are celebrated for their abilities. During a conversation with Mrs Ellers, Horn Man’s mother, she spoke of BHMA being an “island of competence”—a place where people’s various abilities are celebrated rather than drawing focus to their struggles. Through various conversations with students, parents, and staff, as well as through my observations at BHMA and (ultimately) data analysis, I have come to understand that, without having this particular kind of space—where all the young adults living with WS are celebrated for their unique abilities, where they feel safe, supported, appreciated, motivated, engaged and that they belong—the programme would not be as successful.

### 3.3. Strategies: How BHMA Staff Facilitate Life Skills

At BHMA, the staff facilitate the life skills acquisition of young adults living with WS in various ways. The curriculum at BHMA is mostly based on (1) an abilities-first approach, which allows staff to also shift the focus away from the internal struggles that these young adults might be dealing with. The programme is further structured in a way that promotes (2) an experience-first approach while supporting (3) character development among students. BHMA staff also strongly encourage (4) collaborative learning. At BHMA, staff further support students in (5) developing emotional intelligence, while working on their (6) coping skills and (7) fostering hope. Ultimately, BHMA staff want to enable young adults with WS to acquire (8) vocational competence through music.

Liz is very passionate about BHMA’s abilities-first approach and spoke about “celebrating” the unique abilities of every young adult attending BHMA. She also mentioned that “we look at what you can bring to the table and how we can help you”. The music curriculum at BHMA is structured in such a way that the “musical material is carefully chosen, specific to the individuals in the group, to be challenging but attainable”. Staff member David mentioned how he appreciates that he is “able to be part of a team that is constantly ready and willing to break the mould if it benefits our students. Our strength lies in our ability to combine both structure and flexibility”, “while putting the goals and needs of our students first”. One of the key focuses of the BHMA curriculum and strategies is to draw attention away from internal struggles by using music as “a motivating factor to address life skills goals”. Staff members consequently rely on the musical aptitude of young adults with WS and on experience-first approaches to “help develop skills in other areas” because “it’s harder to do it without it”. Throughout the six weeks that I spent at BHMA, I came to realise that the “positive and interactive musical experience” that BHMA offers young adults with WS—“regardless of ability level”—is an integral part of this particular island of competence.

At BHMA, staff members David and Johann consciously facilitate the students finding their place, not only in the ensembles but also in the world, by learning what “values they bring to the group”. It was interesting to see how dance instructor Leanne supported young adults with WS in the Zumba class to find their sense of self. This was especially clear in the case of Jack, who has a strong sense of self. During my visit to BHMA I had the privilege of watching as Tammy, Abe, and Matt’s sense of self and self-esteem developed as a result of the time and attention of BHMA staff during Zumba, individual lessons, and ensemble rehearsals.

Another key goal of BHMA staff is to support young adults with WS in developing their vocational competence. In order to do this, they focus on the development of (1) musical ability, (2) problem-solving skills, (3) career management, and (4) professionalism. When speaking about the goal that BHMA staff have of working towards developing the vocational skills of young adults with WS, Liz said that “being a musician is more than just, you know, singing a great song. There’s a lot else that goes into it, and those basic work skills are really hard”. The increased focus on developing vocational competence among young adults with WS is partly influenced by the higher demand that the world has for these young adults to have a vocation and become more independent. When BHMA staff encourage young adults with WS to set their own goals and make decisions, they ultimately enable them to be successful in their careers. It is also important, however, to discuss the conditions at BHMA that could inhibit the acquisition of life skills for young adults with WS if the staff do not intervene successfully.

### 3.4. Intervening Conditions: Conditions Inhibiting Learning for Young Adults with WS

During my six-week visit to BHMA and during data analysis, I noticed that there are various circumstances that have the potential to inhibit young adults living with WS from successfully learning life skills through music. I was, however, pleasantly surprised at how BHMA staff managed these situations. At BHMA, the degree to which young adults living with WS acquire life skills relies on how the staff manage the fact that many of these young adults tend to have (1) an innate desire to please others, which is possibly related to the perception that young adults with WS often (2) have a low self-esteem. Furthermore, young adults living with WS tend to (3) face specific physical, cognitive, and social challenges, which could explain why they (4) experience negative emotions, which often lead to (5) inappropriate behaviour. Some of the young adults living with WS at BHMA also sometimes struggle with (6) being self-centred, which is sometimes associated with a display of inappropriate behaviour.

Data analysis revealed that, for young adults with WS, “their innate desire to please others blocks their self-realisation” and that they are often “fearful of offending others”. These young adults also tend to struggle with self-esteem, which could be related to the various physical, cognitive, and social challenges that they face daily. One of the BHMA staff members mentioned how young adults with WS have “personality quirks that are huge social challenges for them” and that, although “they bring so much talent and wonderful ideas, and sweet genuineness, they struggle with social skills”. However, during my visit to BHMA and during data analysis, it became clear the staff are sensitive to various conditions that might hinder life skills development for young adults living with WS and that they are able to—through their abilities-first, strengths-based strategies—intervene and assist these young adults in overcoming these challenges. BHMA staff carefully and caringly support these young adults in learning acceptable behaviours which enable them to acquire the life skills they need to live well.

### 3.5. Consequences: Life Skills

The last theoretical concept that I will discuss in this article focuses on the life skills that young adults living with WS learn through music at BHMA, which, in this case, is the consequence of their learning. Data analysis revealed nine life skills that the young adults at BHMA learn through music. Through music, these young adults are able to (1) build their character and (2) connect with others, which consequently supports the development of their (3) emotional intelligence. Engagement in music activities creates opportunities for these young adults to develop (4) coping skills, (5) be hopeful and acquire the necessary skills to become (6) independent. One of the main goals of BHMA staff is to facilitate the development of their (7) vocational competence, which in turn contributes towards them (8) feeling good and ultimately (9) living well.

Through the data analysis I realised that young adults with WS at BHMA often experienced increased self-esteem after a successful learning experience; that they became more self-aware, developed a stronger sense of self, and found their voice. Through music, Allan has been able to “develop his sense of self”. Similarly, music has helped Sizzle to “embrace her strengths and passion for life”. Sizzle supported this notion when she mentioned that “having a disability is just one part of my life”. Thorny Rose, similarly, “speaks freely of having WS and is eager to share traits of the syndrome with others”. During our interview, Liz mentioned how music is “a connector within the WS community, but also a bridge to connect with other disabilities and other people”. Staff member Johann stated that music “changes our relationship fundamentally because we process music very similarly, in most cases. It connects us in a way that other topics or areas of life simply can’t”. I felt a strong sense of togetherness during my visit to BHMA and had the privilege of witnessing how, as Thorny Rose put it, “music brings everybody together” by affording young adults with WS the opportunity to interact and connect with others.

Through engagement in music activities, these young adults are able to develop coping skills by being called up to adapt, deal with conflict, cope with negative emotions, be able to release tension, and draw on music as a lifeline. The ability to adapt to change and unforeseen circumstances is an important skill that these young adults acquire at BHMA through music, and it contributes towards their success, not only during music performances, but also in their vocation of choice. Thorny Rose emphasised that she feels that she “can’t humanly go a day without music” and that she “needs the music”. She explained this by saying that “going without music is like going without water. You can’t do it. You need water every day to stay hydrated”. For me, this must be one of the main motivations behind using music as a means for teaching life skills to young adults with WS. Music truly is an essential part of these young adults’ wellbeing. Sizzle also mentioned how music “helps all of us through the hard times” and that “music is what gave meaning in (her) life”. When young adults with WS learn the life skills required to adapt, deal with conflict and cope with negative emotions, and when music becomes their lifeline, they should be able to be hopeful about their lives and their futures. Through music, young adults with WS at BHMA are able to make sense of their lives and experiences and develop self-actualisation. The music activities at BHMA give these young adults the opportunity to “be who they can be” by developing their character, connecting with others, becoming emotionally intelligent, developing coping skills, learning to be hopeful, becoming independent and vocationally competent, all of which supports them in feeling good about themselves and ultimately living well.

## 4. Discussion: Breaking the Mould: A Theory Explaining How Young Adults Living with Williams Syndrome Learn Life Skills through Music

The theoretical proposition informing the discussion section of this article is:

i. If young adults living with WS have the opportunity to learn through engagement in music activities (central phenomenon) within a safe environment in which they are engaged and feel supported, appreciated and motivated, and feel that they belong and are competent (causal conditions), and;
ii. if educators are willing to focus on the abilities of these young adults by putting their needs and abilities first (strategies);
iii. then the young adults living with WS should be able to overcome various challenges (conditions inhibiting learning) and ultimately develop the life skills they need to live well (consequences).

The lotus flower is used as a metaphor representing the theory presented in this article. This emerging theory was developed by integrating the findings emerging from data analysis with an extensive literature review. The theoretical components (see Section 2, part 2) are therefore determined by the results emerging from this study while an extensive literature review further informs and supports the theoretical components as discussed in the following section. The mud or water from which the flower blooms (symbolically representing difficult times that have to be overcome) represents the conditions inhibiting learning for young adults living with WS. The water that nourishes the flower signifies the central phenomenon, namely engagement in music activities that facilitate learning for young adults living with WS. The leaf on which the flower rests, and which supports and protects the flower, represents the causal conditions required if young adults living with WS are to successfully acquire life skills. The flower leaves represent the strategies that educators need to follow if they are to facilitate the growth of life skills for young adults living with WS. Finally, the pistil, which allows the flower to pollinate and flourish, signifies the life skills that young adults living with WS acquire through engaging in music activities (Figure 2).

### 4.1. Inhibiting Conditions: Specific Challenges Inhibit the Learning of Young Adults Living with WS

WS is defined by certain cognitive, personal, and social challenges [18,64] that could be part of the reasons why therapists and educators often focus on ‘fixing’ the weaknesses of those so diagnosed, rather than building on their strengths [31]. In addition, the various challenges that these young adults face could explain why they experience negative emotions and anxiety [19,65], which often lead to inappropriate behaviour. Furthermore, the personal challenges that young adults with WS experience could influence their ability to foster meaningful relationships as they often struggle to maintain friendships despite their high social drive. These young adults do, however, tend to have an innate desire to please others [17], which could be related to the perception that they often present low self-esteem.

Mastnak and Neuwrithova [65] write that individuals living with WS are often socially vulnerable, as they experience bullying, isolation, abuse, and inconsistent relationships. Sizzle, a young adult with WS attending BHMA, shares her prior experiences of being bullied and feeling isolated through her music compositions and performances. In addition, Mervis and Becerra [18] argue that the challenges that individuals living with WS face regarding pragmatics and communication are often overlooked, which could influence their ability to connect with others. These challenges also influence how young adults living with WS are able to cope and function within their communities. Through the course of studying the literature, collecting, and analysing the data and writing up the findings, it became clear that engagement in music activities provides unique opportunities for these young adults to overcome the cognitive, personal, and social challenges that they face every day. By overcoming these various challenges, these young adults are able to live their lives well and flourish within their communities.

### 4.2. Central Phenomenon: Engagement in Music Activities Facilitates Learning for Young Adults Living with WS

Through data collection and analysis and through studying the scholarly literature, it became clear that engagement in music activities facilitates learning for young adults living with WS [65]. This is consequently also the central phenomenon of the theory that I propose (Figure 2). Although there have been some studies focusing on how music could facilitate learning for individuals living with WS [29,30,31,64,66], as far as I am aware, none specifically focuses on how music supports life skills acquisition. When individuals living with WS engage in music activities, including individual lessons (singing and instrument playing), choir lessons, and movement, they improve their autonomy and ultimately master non-musical goals more successfully. Ioannidi and Samara [31] further suggest that it is necessary to include music in educational frameworks to sufficiently meet the unique learning needs of individuals living with WS. I believe that learning through music is an important aspect to consider regarding life skills acquisition for these young adults, because music is a unique motivator for these young adults and possibly provides opportunities for them to overcome certain cognitive, personal, and social challenges.

Sutela et al. [67] write about how a boy with autism overcame social, personal, and cognitive challenges relating to connection, agency, and focus through engagement in music-and-movement activities. When young adults living with WS and other divergent abilities have a chance to engage in music activities within therapeutic and non-therapeutic contexts, they have an opportunity to develop their identities, become more self-aware, and gain confidence [44,68,69,70,71,72,73]. Music activities could also possibly support young adults with divergent abilities in learning to identify, express, and communicate their emotions, consequently learning how to manage negative emotions and anxiety effectively [45,71,72,73,74], express themselves, find joy, and experience acceptance [65]. Similarly, Saville [72] writes that participation in music activities could support individuals with divergent abilities in addressing challenges such as aggression, isolation, coping with change, and anxiety. Data analysis and a review of the literature revealed that, when young adults with WS learn how to deal with their emotions appropriately, they may also be able to cope with and manage their own negative behaviour [45,72,73,75,76] and connect with others. If music activities are to be utilised as a means for young adults living with WS to successfully learn the life skills that they need to be independent and to flourish, it is important that they have the opportunity to learn within safe, supportive environments.

### 4.3. Causal Conditions: You Are Safe, Valued and Able

Robert Brooks [77] uses islands of competence as a metaphor for hope and strength, as individuals tend to be more hopeful when they believe in their own abilities. Although Brooks [77] coined the metaphor to represent the idea of focusing on the various strengths of children and adults who feel inadequate, helpless, and hopeless, I want instead to borrow this term to describe an environment in which young adults living with WS will be able to flourish. Ioannidi and Samara [31] speak about the importance of the environment in the learning process of children living with WS. They refer to the learning “co-text” [31], p. 271 as a learning environment in which support and communication are key. Garwood and Ampuja [78] argue that intellectually disabled students must feel a sense of connection or belonging within their learning environments, as these students could often easily feel misunderstood, not valued, and be unable to connect with others. Although the environment in which young adults living with WS learn plays a significant role in their learning, the strategies that educators and therapists working with these young adults follow is also vital. If these young adults are to feel supported and able within their learning environments, then parents, educators, and therapists need to prioritise their unique needs and abilities by applying strengths-based teaching strategies.

### 4.4. Strategies: “Breaking the Mould”, Putting Students’ Needs and Abilities-First

A study conducted by Ioannidi and Samara [31] supports the notion of employing strategies that put students’ needs and abilities-first. Through their study, they advocate for the importance of following an abilities-first approach to educating individuals living with WS. They in fact urge educators and therapists to focus on the talents and abilities of individuals living with WS and suggest that there should be room for these individuals to choose the ways in which they want to engage with music in their learning [31]. Reis et al. [29] also write about a music-based talent development approach to educating individuals living with WS and have found that, through music, these individuals were able to acquire skills they possibly would not have been able to develop otherwise. The findings presented in this article reveal that it is essential that young adults living with WS have access to post-secondary education programmes that will enable them to overcome various challenges by developing their abilities and talents, even if that means moving away from traditional teaching methods. The idea of breaking the mould when educating young adults living with WS correlates with findings by Peterson [13], who writes about compassionate education. In his writings, Peterson speaks about compassion as a response to the suffering of others. He further argues that if we are to be compassionate, we need to “recognize and care about the suffering of others” and then act accordingly [13], p. 2. When Hendricks [10] writes about compassionate music teaching, she proposes that teachers support and guide students through a shared enthusiasm for music, but also through supporting students’ desires and passions. Consequently, educators need to prioritise the educational needs of young adults living with WS by providing them with ample opportunities for learning with and from peers, by actively participating in their learning and applying new skills within real-world social settings. I believe that if educators prioritise the needs of young adults living with WS by providing opportunities for collaboration with peers and educators, being compassionate, caring, and open to challenging their own preconceptions, they will lay the foundation for creating optimal conditions for learning in which these young adults could learn the life skills that they need to live well and flourish.

### 4.5. Consequences: Learning Life Skills through Engagement in Music Activities

If young adults living with WS are given opportunities to engage in music activities within safe, supportive environments, and if parents, educators, and therapists put the needs and abilities of these young adults first, they should be able to acquire the life skills that they need to reach their full potential. Music activities could contribute towards these individuals developing the skills associated with expression, autonomy, socialisation, and independence [31]. Similarly, data analysis revealed that the young adults with WS attending BHMA also developed life skills associated with emotional intelligence (emotional expression), self-determination (autonomy), connecting with others (socialisation), and independence. When young adults with WS have the opportunity to develop life skills, including independence, they should be able to live fulfilled lives. Volkman [79] writes that quality of life and the extent to which a person can live a full life are largely influenced by meaningful relationships, identity development, wellbeing, vocation, and one’s ability to appreciate what one has and adapt to change. I contend that young adults living with WS need to cultivate the capacity to be hopeful [80] about the future and develop their ability to reach their goals if they are to live fulfilled lives and flourish [7].

Hewitt [81], when speaking of optimal conditions for developing self-esteem, emphasises that environments that allow people to flourish are paramount for developing self-esteem. He explains that self-esteem depends on how a person feels accepted, secure, and competent, and on whether they can reach their personal goals. Strengths-based teaching strategies could, consequently, enable adolescents to develop the skills associated with building character, confidence, connection, caring, and competence [81]. By developing their competence, talents, and vocational skills through music, young adults living with WS might have the opportunity to feel that they are contributing not only financially to their own and their families’ lives, but are also able to give back to their communities. Furthermore, if these young adults can develop their life skills through music, they should be able to feel that they are valued, feel that they belong [68,71], be hopeful about their futures [80], and have the ability to cope with the challenges of everyday life [44,82]. Ultimately, when young adults living with WS develop life skills through music, they could be empowered to live their best lives.

## 5. Conclusions and Future Research

The theory presented in this article supports advocacy for the inclusion of arts-based methods in education at school and post-secondary levels. This study also draws attention to the importance of applying strengths-based strategies in teaching to meet the unique needs of students with (and without) divergences. Through the course of conducting this study, I was reminded of the importance of letting go of preconceptions and focusing on the abilities and needs of the young adults that we work with [65], while adhering firmly to principles of empathy, care [9,12], compassion [10], wellbeing [7,8,83], mindfulness [84], and social justice. I believe that, for young adults living with WS, the acquisition of life skills through music is paramount to their fulfilled survival in this world. In this regard, subjugated knowledge has relevance. Subjugated knowledge identifies certain types of knowledge that might have been disregarded or deemed less important due to societal power structures [85,86]. These knowledges that are disregarded and deemed less important by majority groups or people in power typically include life experiences and views from people in marginalised groups. Bê [85] writes about subjugated knowledge and people with divergent abilities and mentions how the Western world often disregards how people with various divergences think about, understand, and interact within their communities and the world. Boyce-Tillman [86] argues that power structures in society contribute to the subjugation of those deemed weaker or less important. I contend that this also holds true for young adults living with WS. More research is, however, needed in various contexts studying how young adults living with WS can acquire life skills through music, as this study only draws on data from one site.

We need to acknowledge that the unique learning needs and the importance of music in the learning process of individuals from marginalised groups are not of lesser value. We also need to consider how we can learn from those who have different perspectives when planning curricula or designing our teaching and learning programmes. Researchers, therapists, educators, and policymakers need to advocate for accommodating young adults with WS’s unique educational needs. Consequently, more research is needed that focuses on the needs of young adults with WS through strength-based approaches that place music at the centre of the curricula. Through this study, I came to realise that young adults living with WS do not necessarily have a need to learn academic skills, but rather to engage in music activities, such as being part of various ensembles, individual music lessons, performing for family and friends on a regular basis, having recitals, and performing out in their communities. Through this engagement in music activities, these young adults have the opportunity to strengthen their character development, connect with others, develop emotional intelligence, develop coping skills, be hopeful, become independent, develop vocational skills, feel good about themselves, and live well. We need close collaboration between parents, educators, therapists, teaching institutions, and local businesses, as community engagement is a key aspect in how these young adults acquire life skills. Through engagement in various music activities within a safe, supportive learning environments, where strategies of “breaking the mould” and strengths-based abilities-first approaches are employed, young adults living with WS may be able to overcome various challenges and develop life skills associated with self-actualisation, connection, coping, independence, vocation, and flourishing.

## Data Availability

The data presented in this study are available on request from the corresponding author. The data are not publicly available to ensure the privacy and anonymity of the participants. The results section includes the most significant verbatim quotes from the participants.
